# Banishing “Black/White Thinking”: A Trio of Teaching Tricks

**DOI:** 10.1523/ENEURO.0456-19.2019

**Published:** 2019-12-05

**Authors:** Richard T. Born

**Affiliations:** Department of Neurobiology, Harvard Medical School, Boston, Massachusetts 02115

**Keywords:** counternull, interval statistics, *p* values, significance testing, statistics

## Abstract

Literally hundreds of statisticians have rightly called for an end to statistical significance testing (Amrhein et al., 2019; Wasserstein et al., 2019). But the practice of arbitrarily thresholding *p* values is not only deeply embedded in statistical practice, it is also congenial to the human mind. It is thus not sufficient to tell our students, “Don’t do this.” We must vividly show them why the practice is wrong and its effects detrimental to scientific progress. I offer three teaching examples I have found to be useful in prompting students to think more deeply about the problem and to begin to interpret the results of statistical procedures as measures of how evidence should change our beliefs, and not as bright lines separating truth from falsehood.

## Abstract (precis)

Literally hundreds of statisticians have rightly called for an end to statistical significance testing (Amrhein et al., 2019; Wasserstein et al., 2019). But the practice of arbitrarily thresholding *p* values is not only deeply embedded in statistical practice, it is also congenial to the human mind.

## Significance Statement

I offer specific teaching examples to help students properly think about p values and interval statistics.

## Introduction

Humans are natural born categorizers. We instinctively take continuous variables and draw (often) arbitrary boundaries that allow us to put names to groups. For example, we divide the continuous visible spectrum up into discrete colors like “red,” “yellow,” and “blue.” And the body mass index (BMI) is a continuous measure of a person’s weight-to-height ratio, yet a brief scan of the Internet turns up repeated examples of the classification shown in Table 1.

**Table 1:** Classification of BMI

BMI	Category
<18.5	Underweight
18.5–24.9	Normal or healthy weight
25.0–29.9	Overweight
>30	Obese

Source: Centers for Disease Control and Prevention.

In some cases, such as for color, certain categories appear to be “natural,” as if they were baked into our brains (Rosch, 1973). In other cases, categorization is related to the need to make decisions, as is the case for many medical classifications. And the fact that we communicate our ideas using language—words being discrete entities—surely contributes to this tendency.

Nowhere is the tendency more dramatic—and more pernicious—than in the practice of null hypothesis significance testing (NHST), based on *p* values, where an arbitrary cutoff of 0.05 is used to separate “truth” from “falsehood.” Let us set aside the first obvious problem that in NHST we never accept the null (i.e., proclaim falsehood) but rather only fail to reject it. And let us also ignore the debate about whether we should change the cutoff to something more stringent, say 0.005 (Benjamin et al., 2018), and instead focus on what I consider to be the real problem: the cutoff itself. This is the problem I refer to as “black/white thinking.”

Because this tendency to categorize using *p* values is (1) natural and (2) abundantly reinforced in many statistics courses, we must do more than simply tell our students that it is wrong. We must show them why it is wrong and offer better ways of thinking about statistics. What follows are some practical methods I have found useful in classroom discussions with graduate students and postdoctoral fellows in neuroscience.

## Example 1

**Figure 1.**
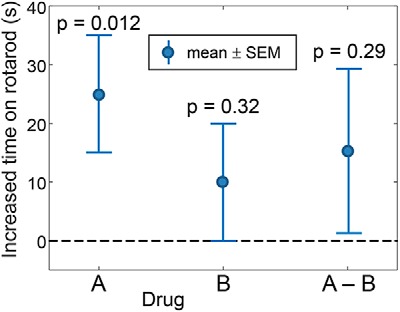
Efficacy of two different drugs in increasing the time that mice can remain on a rotating rod. For each drug, the mean effect and the associated SEs are shown. The dashed line represents the null value of no difference. Example taken from Gelman and Stern (2006).

In class, I start with an example of a statistical error that is known to be extremely common in the neuroscience literature (Nieuwenhuis et al., 2011). I took the numbers directly from the classic article by Gelman and Stern (2006) that has one of my all-time favorite titles: “The Difference Between ‘Significant’ and ‘Not Significant’ is not Itself Statistically Significant.” In this made-up example (Fig. 1), we compare two drugs being tested for their efficacy in increasing the time a genetic mouse model of amyotrophic lateral sclerosis can remain on a rotating rod. Drug A, on average, increases performance by 25 s with an SE of 10 s—what most people would categorize as a “statistically significant effect” (*p* = 0.012). Drug B, on the other hand, is not even close (effect size, 10 ± 10 s; *p* = 0.32).

Before showing the data for the direct contrast in Figure 1 (A–B), I ask the class the simple question of whether, based on the data shown on the left part of Figure 1, we can conclude that drug A is “significantly better” than drug B at increasing performance. I encourage them to first discuss the question with their immediate neighbors, and then I ask them to vote “yes” or “no,” either with a show of hands or, if available, some form of a clicker response. In general, the vast majority of the class votes yes, but there is always some visible trepidation (e.g., sheepish hand raising), since the students figure that there must be a trick if I am asking them something so apparently obvious.

I then display the contrast shown on the right side of Figure 1 as “A–B.” A direct comparison of the two drugs reveals a difference of 15 s with an SE of 14, and a corresponding *p* value of 0.29. So I ask them, “How can this be? One drug ‘works’ and the other drug does not, so there must be a difference, right?” This generates some murmuring among the class, and this is an excellent opportunity for a discussion of “What’s going on here?” At some point, I usually interject that, given any two drugs with any two nonequal *p* values, I can set a criterion that makes one of the drugs “work” and the other not. This helps point out the arbitrary nature of any *p* value cutoff and the major error of interpreting the failure to reject the null hypothesis (H0) for one of the drugs as not working, as well as the important idea that we want to make our inference about differences between the drugs based on the difference A–B.

I close off this example by displaying the title of the article by Gelman and Stern (2006), and I encourage the students to repeat the title as a mantra each night before they go to bed and each morning when they awake for the next 2 months. And I add in a favorite quote from Rosnow and Rosenthal (1989): “That is, we want to underscore that, surely, God loves the 0.06 nearly as much as the 0.05. Can there be any doubt that God views the strength of evidence for or against the null as a fairly *continuous* function of the magnitude of *p*?” (emphasis added). As this quote is bang on and moderately funny, it puts a memorable cap on the exercise.

## Example 2

**Figure 2.**
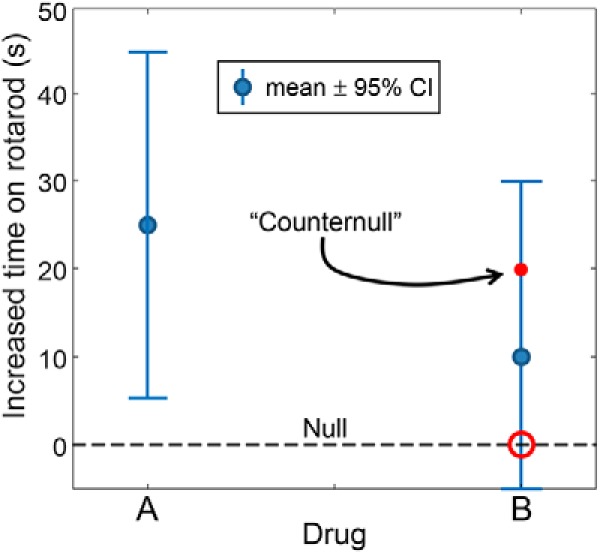
Same data as for Figure 1, except that SE bars have been replaced with 95% confidence intervals. The counternull is the value of the effect size that is equidistant from the mean (vs the null), but on the opposite side. See Rosenthal and Rubin (1994).

From Figure 1, there is an easy segue to the second concept I find useful, that of the “counternull,” first described by Rosenthal and Rubin (1994). To do this, I simply replace the SE bars with 95% confidence intervals (CIs; Fig. 2), which clearly shows why the null was not rejected for drug B: the 95% CI contains the null value of zero. But Rosenthal and Rubin (1994) would also have us consider the point that is equally distant from the mean but on the opposite side: this is the value they refer to as the counternull. Simply put, it is that value of the effect size (in our example, a performance increase of just >20 s) that is supported by exactly the same amount of evidence as the null value.

I like this statistic, because it gets the students thinking about more of the confidence interval than just whether or not it contains the null value (i.e., NHST). I ask them, would an effect size of this magnitude be of behavioral significance? If so, we might not be so quick to give up on drug B. We can certainly see that there is a broad range of plausible effect sizes that would be beneficial (as well as some that would be detrimental).

This also presents a good opportunity to start a discussion about how one might decide which of two drugs to take. Is statistical significance a good criterion? By simply rescaling the axes, one could show a strongly significant effect for a change in performance that would be negligible in terms of behavioral benefit—so the actual effect size, and not just its *p* value, matter. And what if we were told that drug A has a high incidence of toxic side effects? Or that it needed to be taken by a twice-daily intravenous injection?

One additional issue that can be brought up here is how we write statistical results in our articles. If we banish from our students’ lexicons the phrase “statistically significant,” what do we give them as a replacement? My practice is to encourage them to always include a point estimate of the effect size, generally the mean, along with a 95% confidence interval—a practice that is increasingly recommended by neuroscience journals (Calin-Jageman and Cumming, 2019), including *eNeuro*. In certain cases where the null value is included within the 95% CI, it might be useful to include the counternull, particularly when its magnitude would represent an important biological effect.

## Example 3

**Figure 3.**
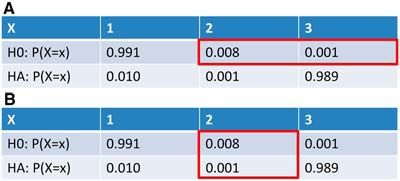
Each table shows the probabilities with which a random variable, *X*, can take on values of 1, 2, or 3 under two different hypotheses. ***A***, The red box shows the Frequentist perspective in which only the H0 probabilities are considered and the most powerful test is to reject H0 for *X* = 2 or 3. ***B***, A more Bayesian perspective is shown. Example is from Johnson (2013a), who apparently borrowed it from Berger and Wolpert (1988).

My third example is taken from an interesting article by Johnson (2013a), and it is designed to nudge the students toward a Bayesian perspective (Fig. 3).

In this example, we are given a single datum, namely that *X* = 2 and are asked to make an inference about the distribution from which it was drawn. A good Frequentist (Fig. 3*A*) would look at the probabilities under H0 and determine that she should reject H0 for *X* = 2 or 3, as this would give a *p* value of <0.01. However, a Bayesian would compare the values in the red box of Figure 3*B* and realize that, for *X* = 2, H0 is eight times more likely than HA (a Bayes factor). In fact, a simple calculation using Bayes’ rule (which I do on the whiteboard) and assuming that the two hypotheses are a priori equally likely, reveals that the posterior probability that H0 is true is 0.89, although our Frequentist has confidently rejected it at *p* < 0.01 (“highly significant!”). This effectively creates a tension between what common sense tells us is the better approach and what the students have long held to be the right way to think.

Apart from this heavily rigged example, why is a Bayesian perspective helpful in combating black/white thinking? Well, the spirit of Bayesian data analysis is exactly what we want to inculcate in our students: using our experimental data, via the likelihood, to inform us how much we should change our beliefs. It encourages the better interpretation that the results of experiments should change our beliefs about hypotheses in a continuous way and not be used to draw sharp lines between truth and falsehood. This is not to say that Bayesian thinking is a panacea—one can create thresholds with Bayes factors as surely as one can with *p* values, and it is the threshold setting that is the problem. So what I try to communicate to my students is that we will continue to publish and perish in a largely Frequentist world for some time (Efron, 2013), but it behooves us all to be more Bayesian in spirit. And it even appears that Bayesian analyses may be on an upward trend in the neurosciences (Boekel et al., 2015).

**Figure 4.**
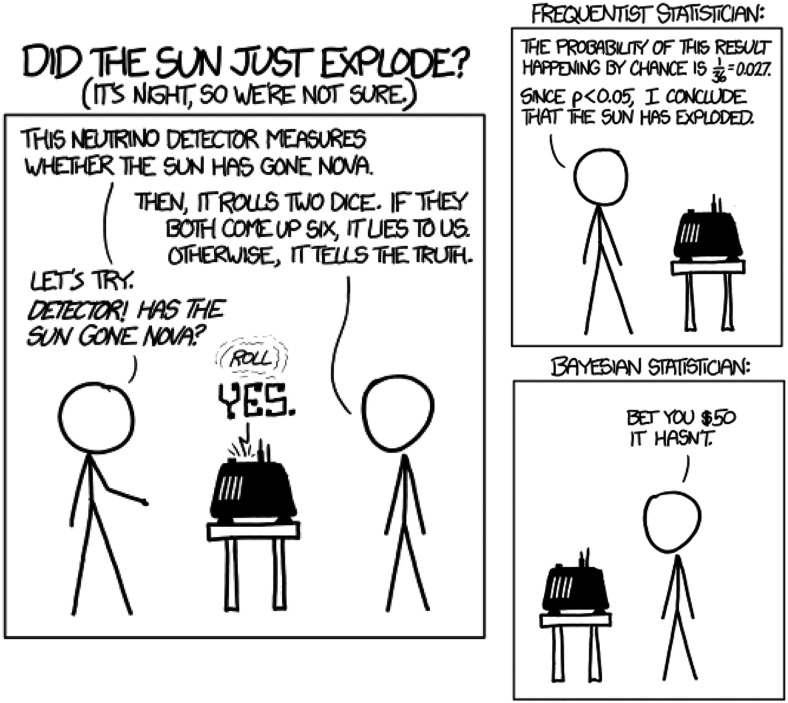
The importance of the prior probability of H0 when evaluating *p* values. Source: xkcd (https://xkcd.com/1132/).

Finally, the introduction of Bayes’ rule allows us to address another critical shortcoming of NHST by considering priors. While this is a thorny topic when approached broadly, a narrower consideration of the prior probability of H0 is useful when considering, for example, “ground-breaking experiments” that are proffered with no more evidence than “*p* < 0.05.” I introduce this problem with my favorite xkcd cartoon [Fig. 4 (see https://xkcd.com/1132/)].

If we start with the belief that it is extremely unlikely for the sun to explode in any small interval of time, then we will not be persuaded by such flimsy evidence as *p* < 0.03. This is a nice illustration of the LaPlacian notion that extraordinary claims require extraordinary evidence. From this perspective, the exercise of converting *p* values to minimum Bayes’ factors (Goodman, 2001) and then applying Bayes’ rule to different scenarios of prior probability (Nuzzo, 2014) can be eye opening for the students. A *p* value just under 0.05 does not push us as far away from H0 as we would like to think it does (Johnson, 2013b).

And in the spirit of closing with a memorable quote, I share a favorite exclamation of one of my early mentors, David Hubel, whenever I approached him with some claim that struck him as highly implausible: “That’s the kind of result you wouldn’t believe even if it were true!” For some time, this statement bothered me a lot—was this great scientist scoffing at evidence?—until I realized that it reflected a Bayesian perspective combined with a characteristically deep awareness of the brittleness of a *p* value criterion for “truth.”

## Discussion

There is a broad consensus among statisticians that significance testing based on *p* values is bad statistical practice. Moreover, this consensus has existed for many years (Wasserstein et al., 2019). So why does the practice persist so stubbornly? I have argued that it is not just inertia in the teaching and practice of statistics, but that it also stems from our natural proclivity to sort continuous data into clean classes to which we can give names—what I have called black/white thinking. Because of this tendency, we need to work harder and be more creative in teaching our students better ways of thinking. I have offered here several specific teaching examples (including PowerPoint slides; Extended Data Fig. 1-1) that I have found useful in this regard. I hope they will be added to by others.

10.1523/ENEURO.0456-19.2019.f1-1 Figure 1-1 PowerPoint slides used to teach these examples (and a few others). Download Figure 1-1, PPTX file.

## Synthesis

Reviewing Editor: Christophe Bernard, INSERM & Institut de Neurosciences des Systèmes Decisions are customarily a result of the Reviewing Editor and the peer reviewers coming together and discussing their recommendations until a consensus is reached. When revisions are invited, a fact-based synthesis statement explaining their decision and outlining what is needed to prepare a revision will be listed below.

Both your reviewer Bob Calin-Jageman and I really enjoyed this paper.

I copy Bob's comments, with which I agree: you have a nice set of examples that lucidly explain some important caveats to interpreting p values. It is pitched mostly as teaching exercises, but I'm confident even seasoned researchers could benefit from reading this short paper and reflecting on the examples.

One question was the relevance to the neuroscience field. Conceptually, the paper fits in the educational mission of eNeuro. You may consider trying to connect with the conversation about p values within neuroscience. Specifically:

* The examples aren't based on neuroscience. Superficial, but it can really help researchers and students think about analyses when couched in familiar research contexts.

* You could better connect to the ongoing discussion of p values and their pitfalls in neuro.

** For example, you clearly describe the problem of checking for differences by evaluating significance status. This is something that's been specifically analyzed within the neuroscience literature (https://www.nature.com/articles/nn.2886).. so it would be useful to cite that paper, which shows that this error is actually common in the published neuroscience literature.

** You mention the utility of Bayesian perspectives.. might be worth mentioning that this is a growing trend in neuroscience (https://www.ncbi.nlm.nih.gov/pubmed/25684445)

** Finally, eNeuro has recently amended its authorship guidelines to encourage authors to report effect sizes with uncertainty estimates (https://www.eneuro.org/content/6/4/ENEURO.0205-19.2019/tab-article-info). You use CIs to help dispel misconceptions that arise from p values, so that's another possible point of connection.

Always signed:

## Author Response

Thank you very much for the positive and extremely helpful reviews. Below I include the original comments in Calibri font, while my responses are in Times New Roman and initiated with a boldface “Response:”.

Synthesis of Reviews:

Significance Statement Comments for Author (Required):

N/A

Comments on the Visual Abstract for Author (Required):

N/A

Synthesis Statement for Author (Required):

Both your reviewer Bob Calin-Jageman and I really enjoyed this paper.

I copy Bob's comments, with which I agree: you have a nice set of examples that lucidly explain some important caveats to interpreting p values. It is pitched mostly as teaching exercises, but I'm confident even seasoned researchers could benefit from reading this short paper and reflecting on the examples.

One question was the relevance to the neuroscience field. Conceptually, the paper fits in the educational mission of eNeuro. You may consider trying to connect with the conversation about p values within neuroscience. Specifically:

* The examples aren't based on neuroscience. Superficial, but it can really help researchers and students think about analyses when couched in familiar research contexts.

Response: I have modified the example in figures 1 and 2 to be more neuroscience-y. Note that in the original paper (Gelman & Stern 2006), only the numbers are given-that is, abstract “effect estimates” with the mean and standard error for each of two studies. I made up the blood pressure example on-the-fly during a lecture I was giving to a group of postdocs from departments across the medical school (not just neurobiology), and I figured that anyone can relate to drugs for treating blood pressure. So I simply replaced the blood-pressure story with two drugs being tested on a mouse model of amyotrophic lateral sclerosis. I had originally noted the “made up” aspect of the example in a footnote, but this was not allowed by eNeuro style rules.

Note that I could pretty easily find a real example from the neuroscience literature (especially given the new pointer to the paper by Nieuwenhuis et al. 2011 (see below). However, I prefer to go with the artificial example for two reasons. First, I find it awkward to call out actual, specific mistakes of colleagues in this kind of “friendly” teaching commentary. Second, I somehow like the idea of paying homage to Gelman & Stern (2006) by “quoting” them exactly.

Since example #3 is purely abstract and rigged to make a specific point, I'm not sure it would benefit from embedding in a neuroscience context. I have currently left this one as is, but I'd happily change it if one of you has a better idea.

Example #4 is too perfect as is. I wouldn't dare to mess with xkcd!

* You could better connect to the ongoing discussion of p values and their pitfalls in neuro.

** For example, you clearly describe the problem of checking for differences by evaluating significance status. This is something that's been specifically analyzed within the neuroscience literature (https://www.nature.com/articles/nn.2886).. so it would be useful to cite that paper, which shows that this error is actually common in the published neuroscience literature.

Response: This is fantastic! I had forgotten about this paper, and it perfectly supports how common a mistake this is. Thank you. I've added this reference at the very beginning of the description of example #1:

“In class, I start with an example of a statistical error that is known to be extremely common in the neuroscience literature (Nieuwenhuis et al. 2011).”

** You mention the utility of Bayesian perspectives.. might be worth mentioning that this is a growing trend in neuroscience (https://www.ncbi.nlm.nih.gov/pubmed/25684445)

Response: I have added a sentence to indicate this and included the reference:

“So what I try to communicate to my students is that we will continue to publish and perish in a largely Frequentist world for some time (Efron 2013), but it behooves us all to be more Bayesian in spirit. And it even appears that Bayesian analyses may be on an upward trend in the neurosciences (Boekel et al. 2015).”

** Finally, eNeuro has recently amended its authorship guidelines to encourage authors to report effect sizes with uncertainty estimates (https://www.eneuro.org/content/6/4/ENEURO.0205-19.2019/tab-article-info). You use CIs to help dispel misconceptions that arise from p values, so that's another possible point of connection.

Response: Thank you for the reference. I have added it to the paragraph where I talk about how to write up results:

“One additional issue that can be brought up here is how we write statistical results in our papers. If we banish from our students' lexicons the phrase “statistically significant,” what do we give them as a replacement? My practice is to encourage them to always include a point estimate of the effect size, generally the mean, along with a 95% confidence interval-a practice that is increasingly recommended by neuroscience journals (Calin-Jageman & Cumming 2019), including eNeuro.”
