# Supplementary material for: Banishing “Black/White Thinking”: A Trio of Teaching Tricks
Source: eNeuro. 2019 Dec 5;6(6):ENEURO.0456-19.2019. doi: 10.1523/ENEURO.0456-19.2019 (PMC6900463; doi:10.1523/ENEURO.0456-19.2019)
Supplement: Figure 1-1 — PowerPoint slides used to teach these examples (and a few others). Download Figure 1-1, PPTX file. [file sup_enu-eN-COM-0456-19-s02.pptx]

## Slide 1
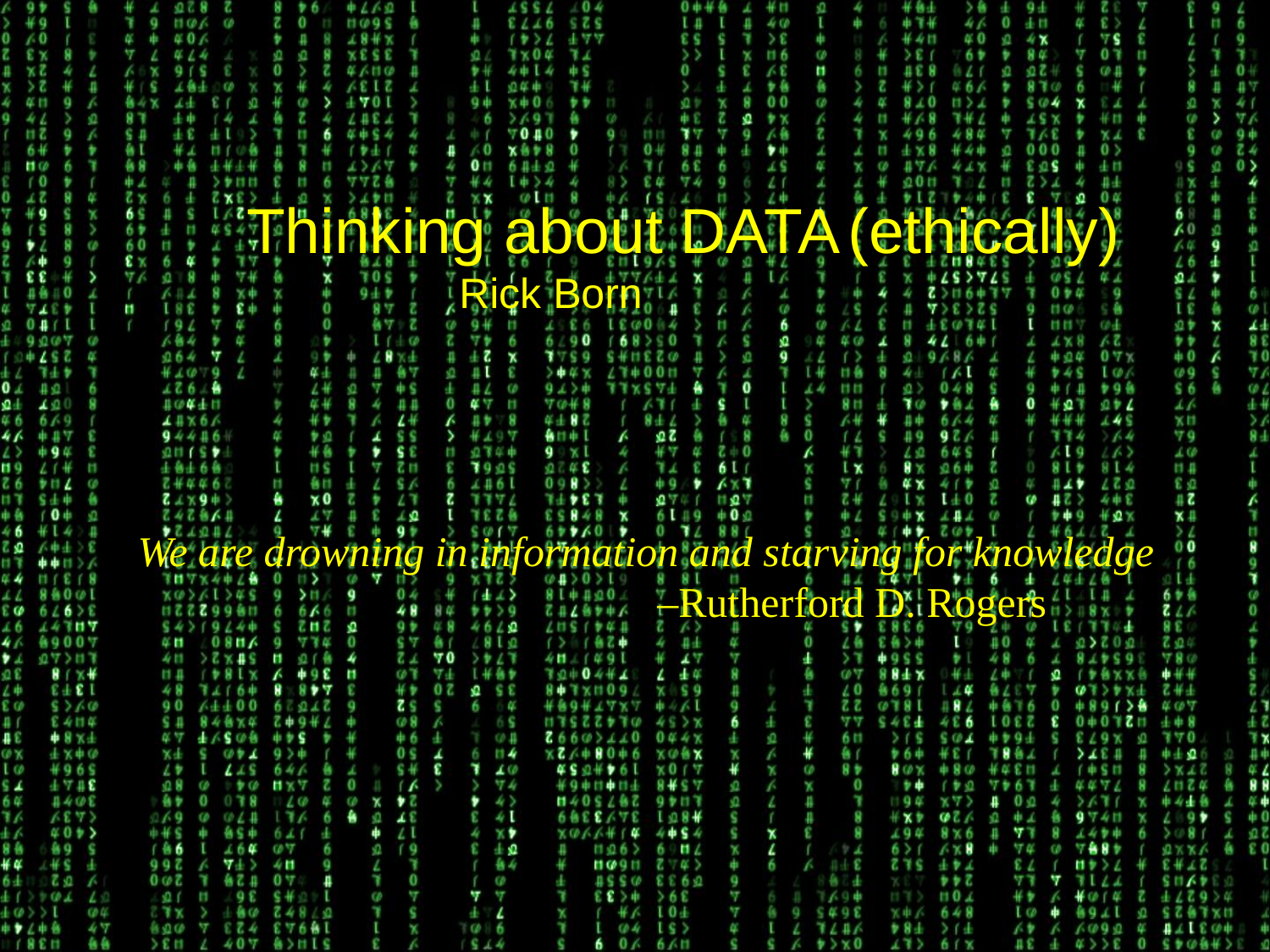

Thinking about DATA
Rick Born
(ethically)
We are drowning in information and starving for knowledge
			 –Rutherford D. Rogers

## Slide 2
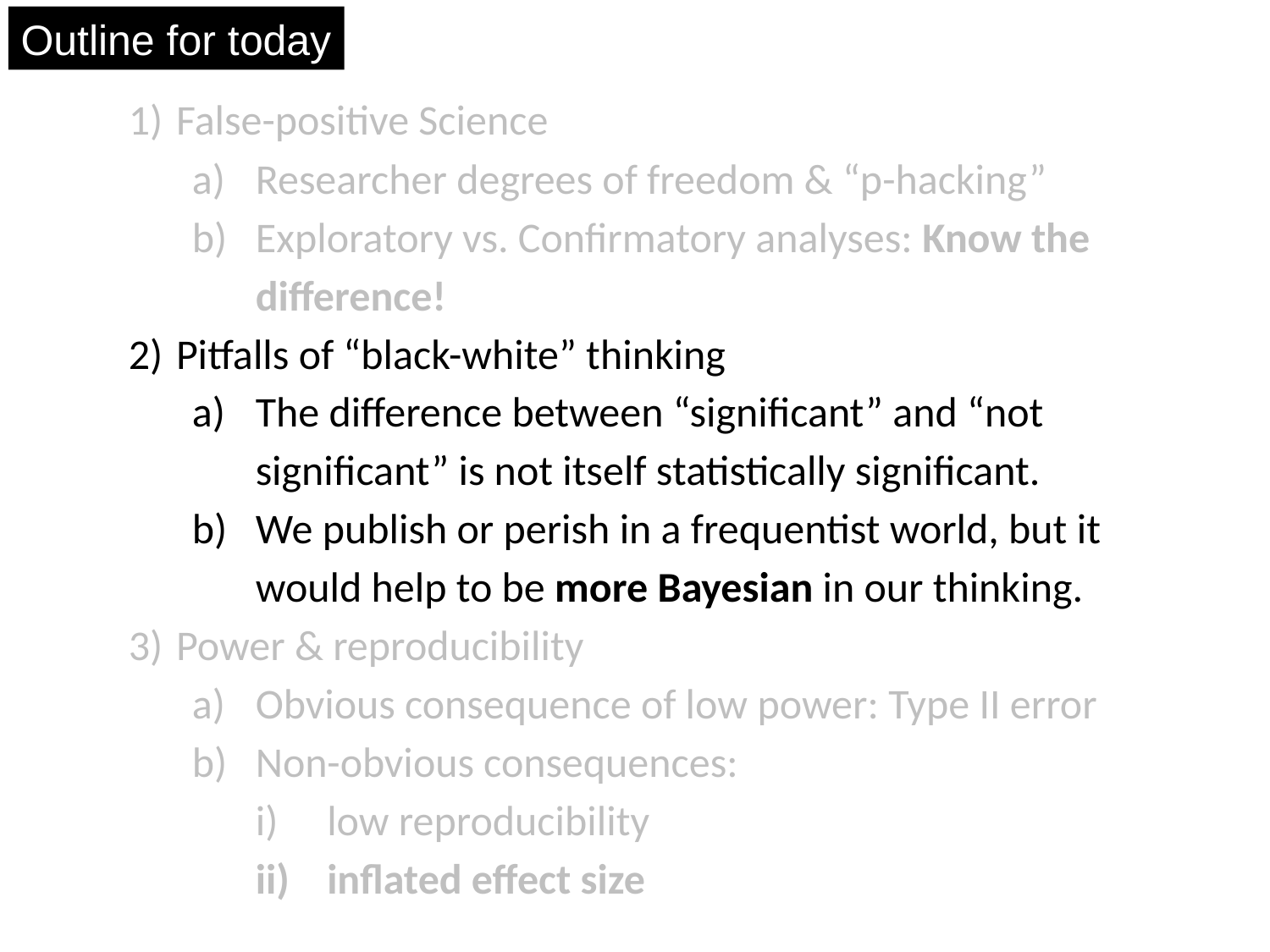

Outline for today
False-positive Science
Researcher degrees of freedom & “p-hacking”
Exploratory vs. Confirmatory analyses: Know the difference!
Pitfalls of “black-white” thinking
The difference between “significant” and “not significant” is not itself statistically significant.
We publish or perish in a frequentist world, but it would help to be more Bayesian in our thinking.
Power & reproducibility
Obvious consequence of low power: Type II error
Non-obvious consequences:
low reproducibility
inflated effect size

## Slide 3
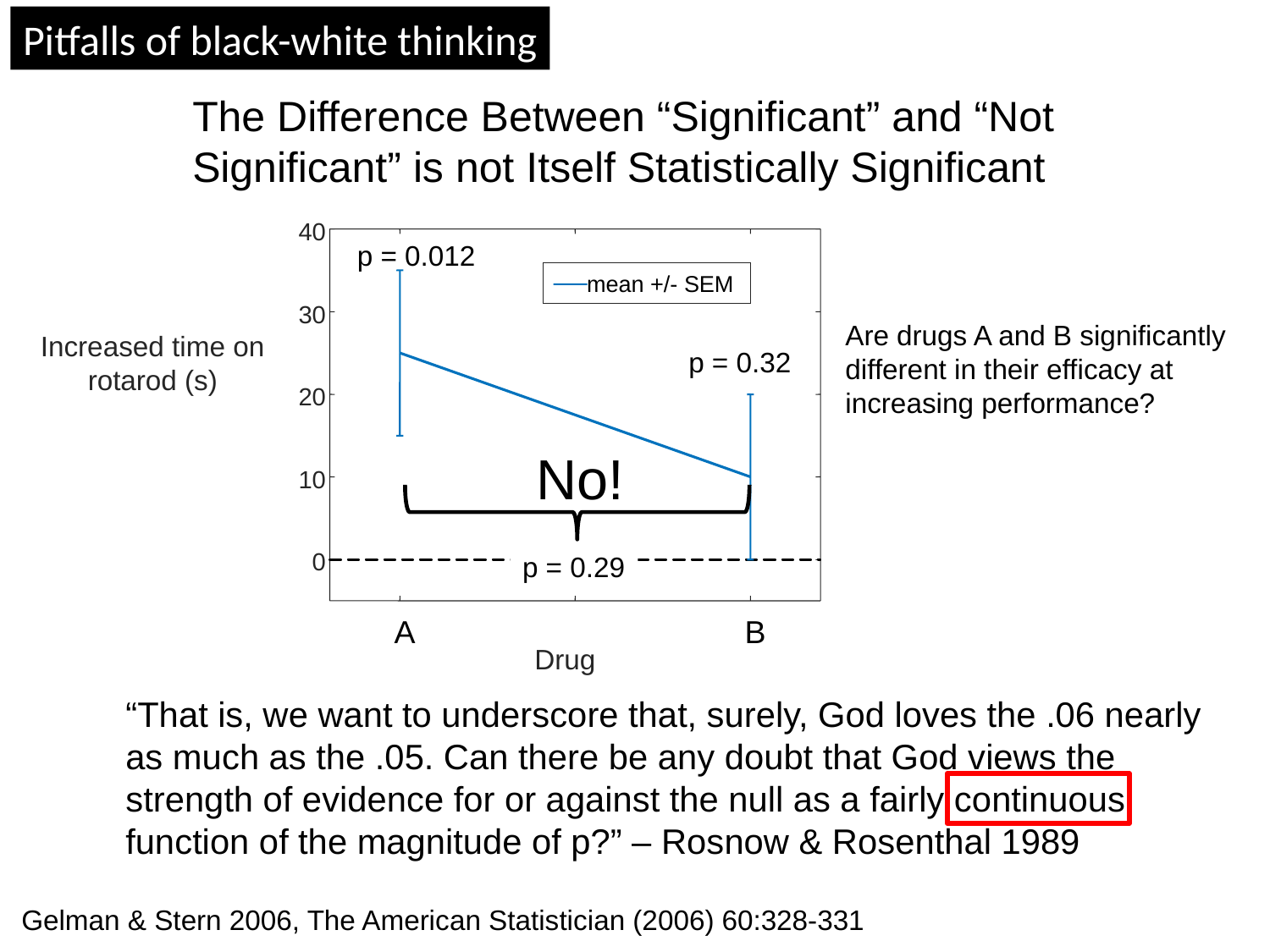

Pitfalls of black-white thinking
The Difference Between “Significant” and “Not Significant” is not Itself Statistically Significant
40
30
20
10
0
p = 0.012
mean +/- SEM
Are drugs A and B significantly different in their efficacy at increasing performance?
Increased time on rotarod (s)
p = 0.32
No!
p = 0.29
A
B
Drug
“That is, we want to underscore that, surely, God loves the .06 nearly as much as the .05. Can there be any doubt that God views the strength of evidence for or against the null as a fairly continuous function of the magnitude of p?” – Rosnow & Rosenthal 1989
Gelman & Stern 2006, The American Statistician (2006) 60:328-331

## Slide 4
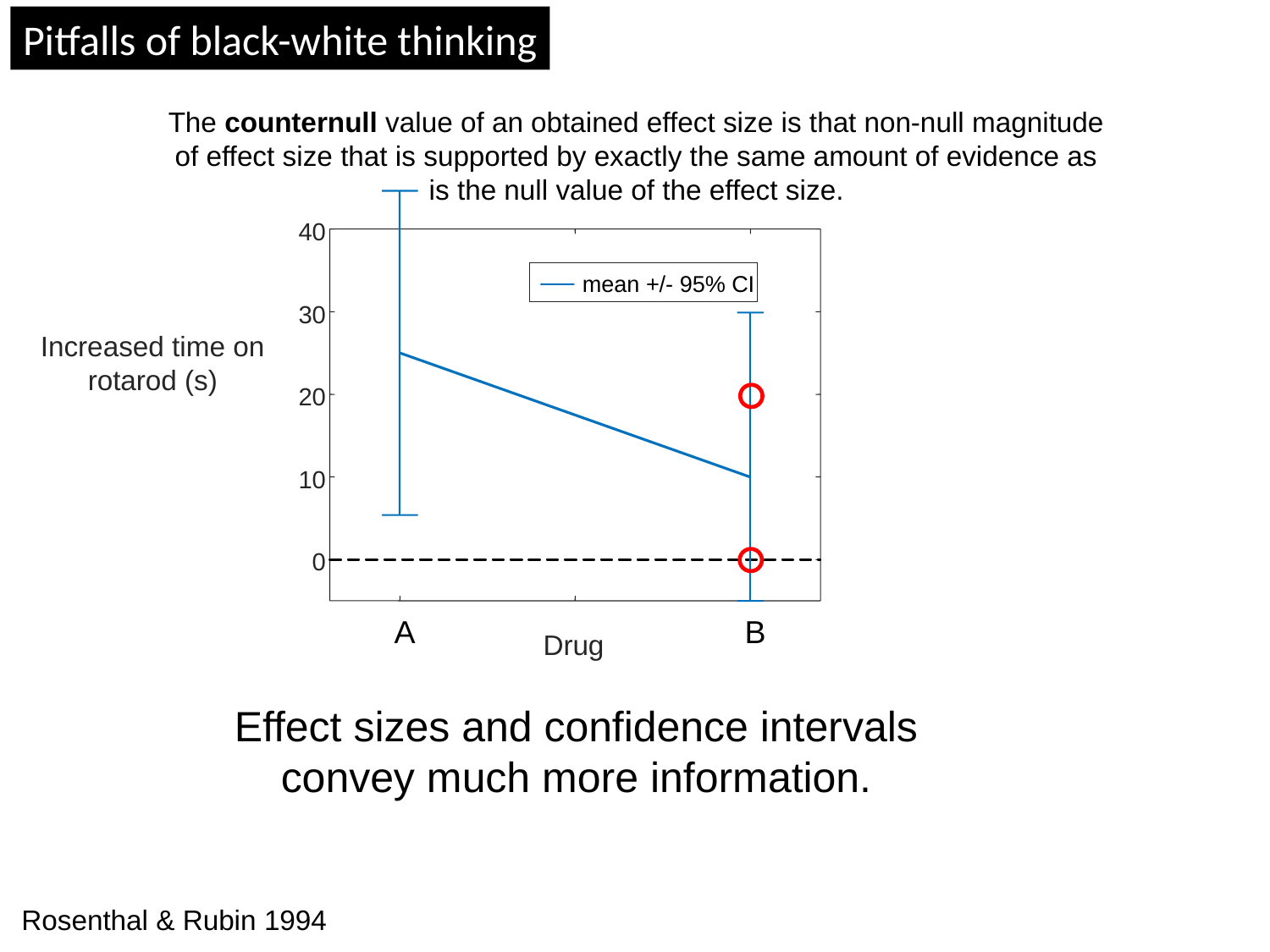

Pitfalls of black-white thinking
The counternull value of an obtained effect size is that non-null magnitude of effect size that is supported by exactly the same amount of evidence as is the null value of the effect size.
40
30
20
10
0
mean +/- 95% CI
Increased time on rotarod (s)
A
B
Drug
Effect sizes and confidence intervals
convey much more information.
Rosenthal & Rubin 1994

## Slide 5
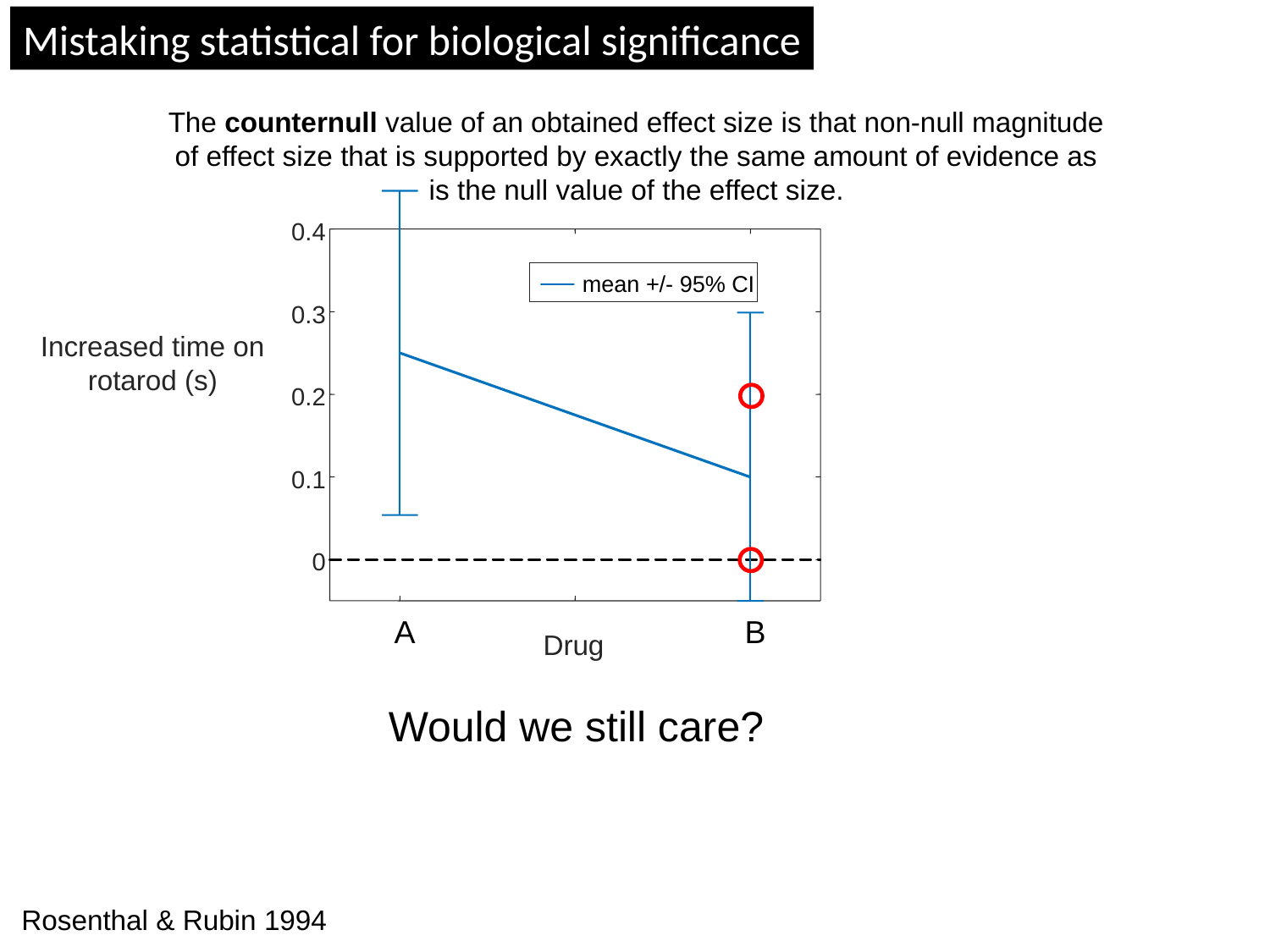

Mistaking statistical for biological significance
The counternull value of an obtained effect size is that non-null magnitude of effect size that is supported by exactly the same amount of evidence as is the null value of the effect size.
0.4
0.3
0.2
0.1
0
mean +/- 95% CI
Increased time on rotarod (s)
A
B
Drug
Would we still care?
Rosenthal & Rubin 1994

## Slide 6
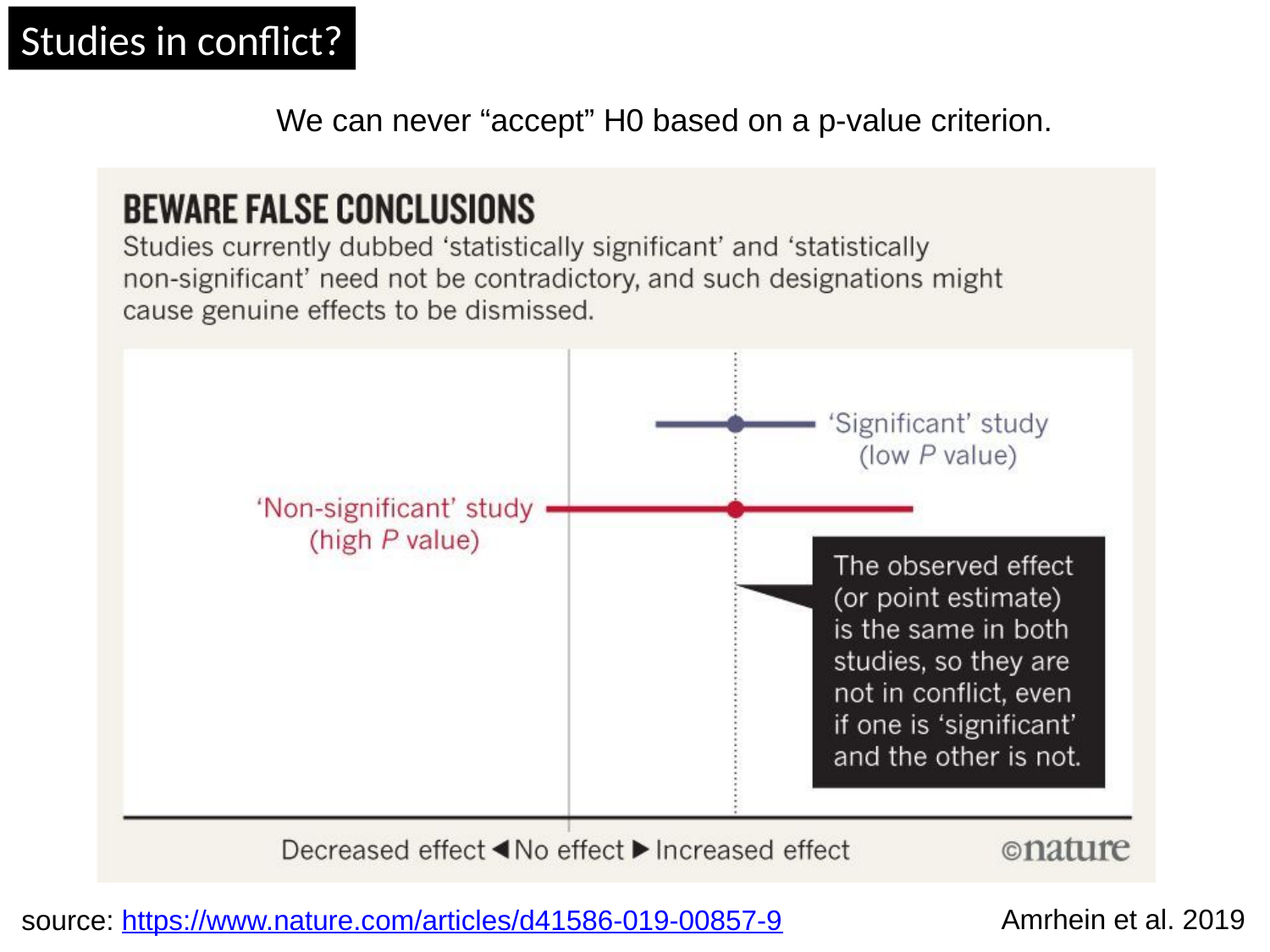

Studies in conflict?
We can never “accept” H0 based on a p-value criterion.
Amrhein et al. 2019
source: https://www.nature.com/articles/d41586-019-00857-9

## Slide 7
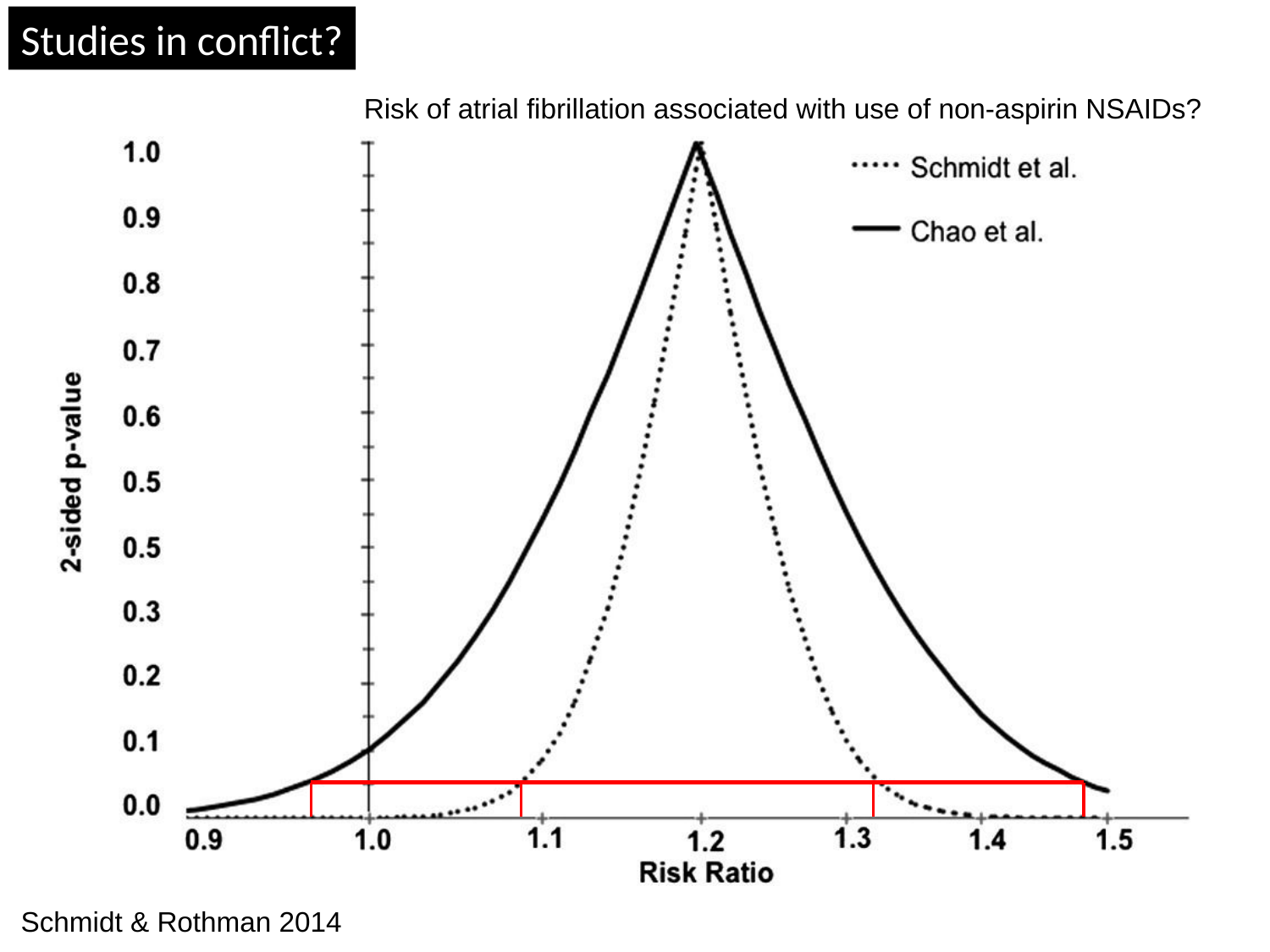

Studies in conflict?
Risk of atrial fibrillation associated with use of non-aspirin NSAIDs?
Schmidt & Rothman 2014

## Slide 8
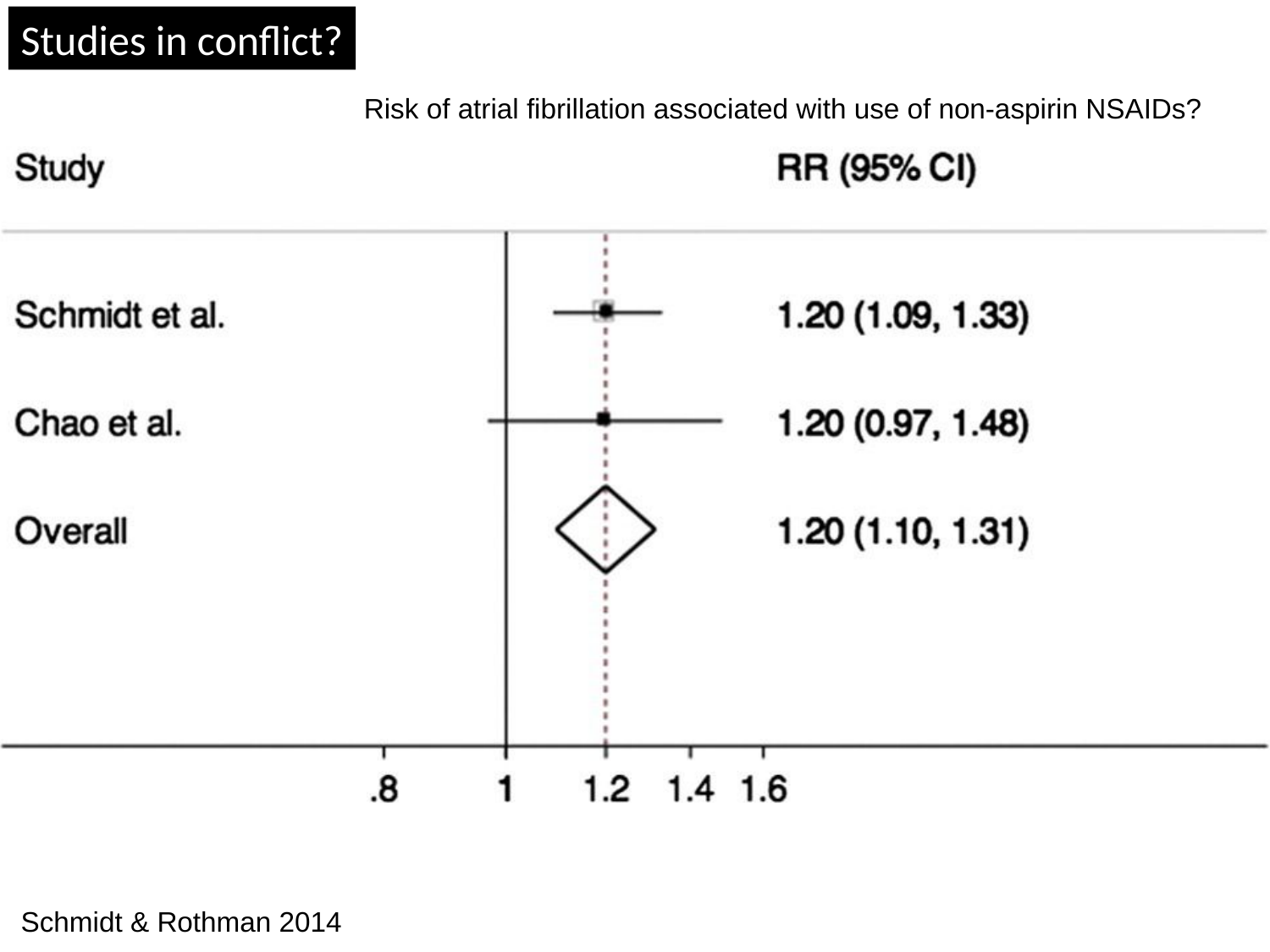

Studies in conflict?
Risk of atrial fibrillation associated with use of non-aspirin NSAIDs?
Schmidt & Rothman 2014

## Slide 9
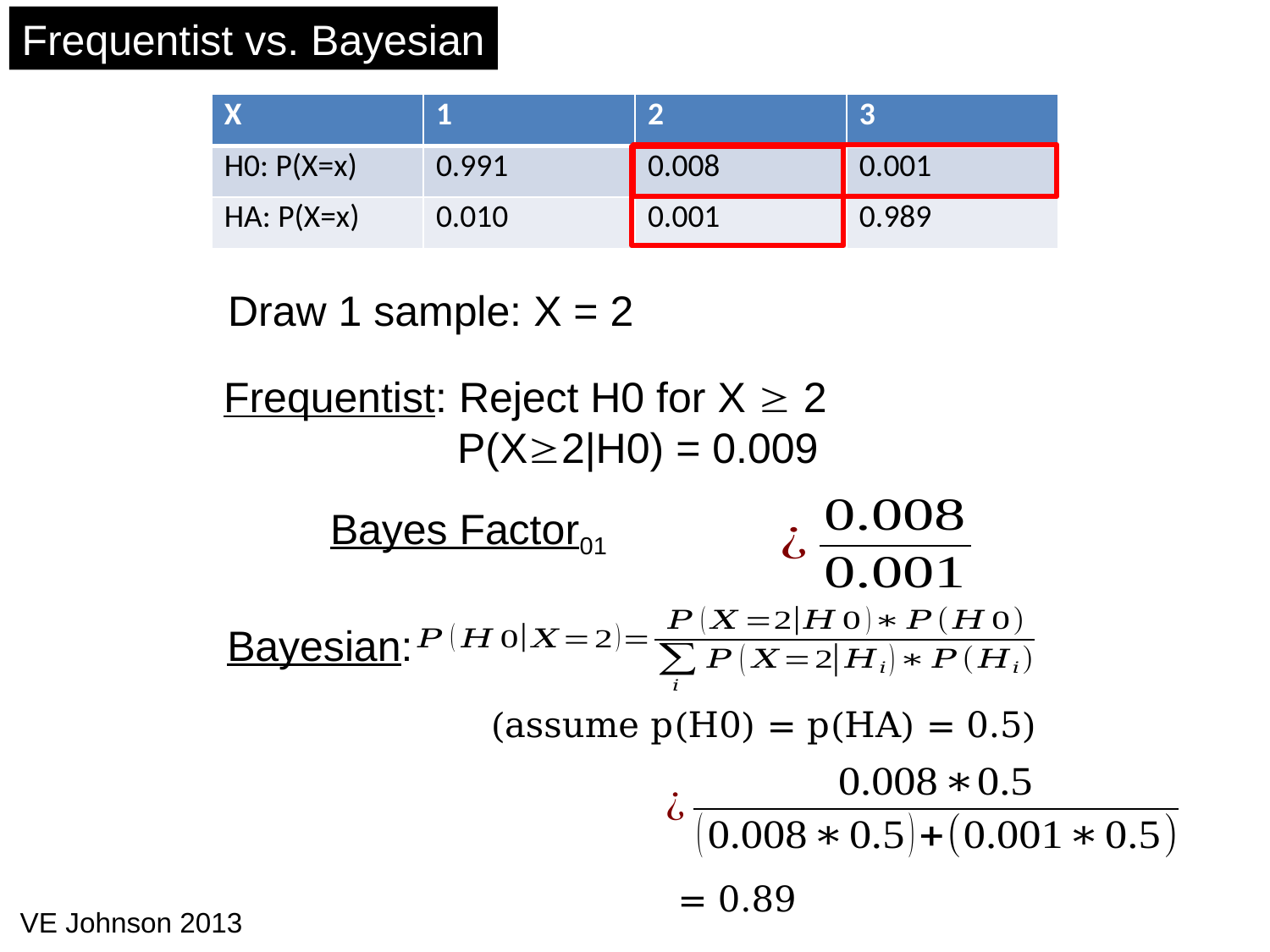

Frequentist vs. Bayesian
| X | 1 | 2 | 3 |
| --- | --- | --- | --- |
| H0: P(X=x) | 0.991 | 0.008 | 0.001 |
| HA: P(X=x) | 0.010 | 0.001 | 0.989 |
Draw 1 sample: X = 2
Frequentist: Reject H0 for X ≥ 2
	 P(X≥2|H0) = 0.009
Bayesian:
(assume p(H0) = p(HA) = 0.5)
= 0.89
VE Johnson 2013

## Slide 10
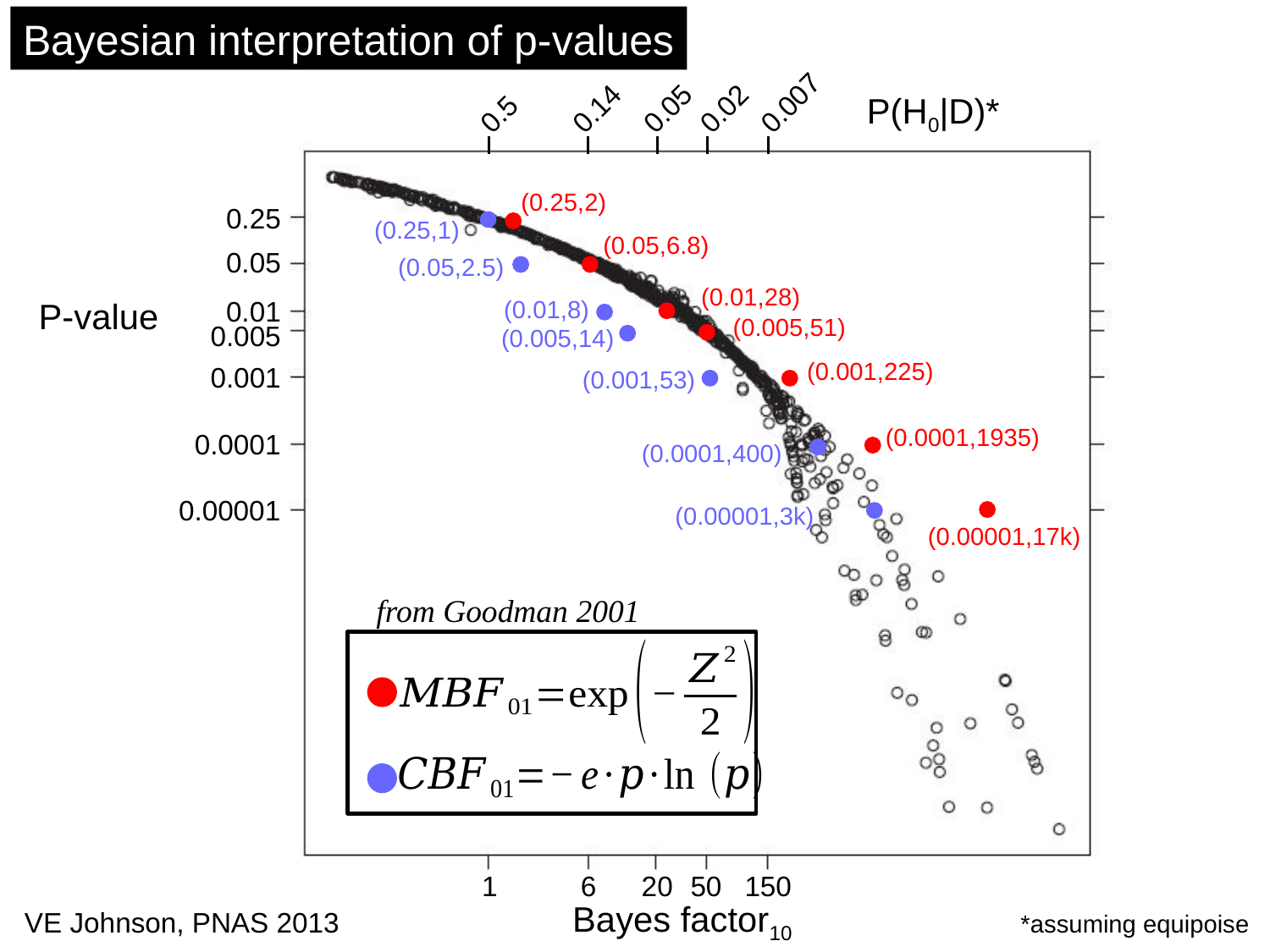

Bayesian interpretation of p-values
0.007
P(H0|D)*
0.14
0.05
0.02
0.5
(0.25,2)
0.25
0.05
0.01
0.005
0.001
0.0001
0.00001
(0.25,1)
(0.05,6.8)
(0.05,2.5)
(0.01,28)
(0.01,8)
P-value
(0.005,51)
(0.005,14)
(0.001,225)
(0.001,53)
(0.0001,1935)
(0.0001,400)
(0.00001,3k)
(0.00001,17k)
from Goodman 2001
1
6
20
50
150
Bayes factor10
VE Johnson, PNAS 2013
*assuming equipoise

## Slide 11
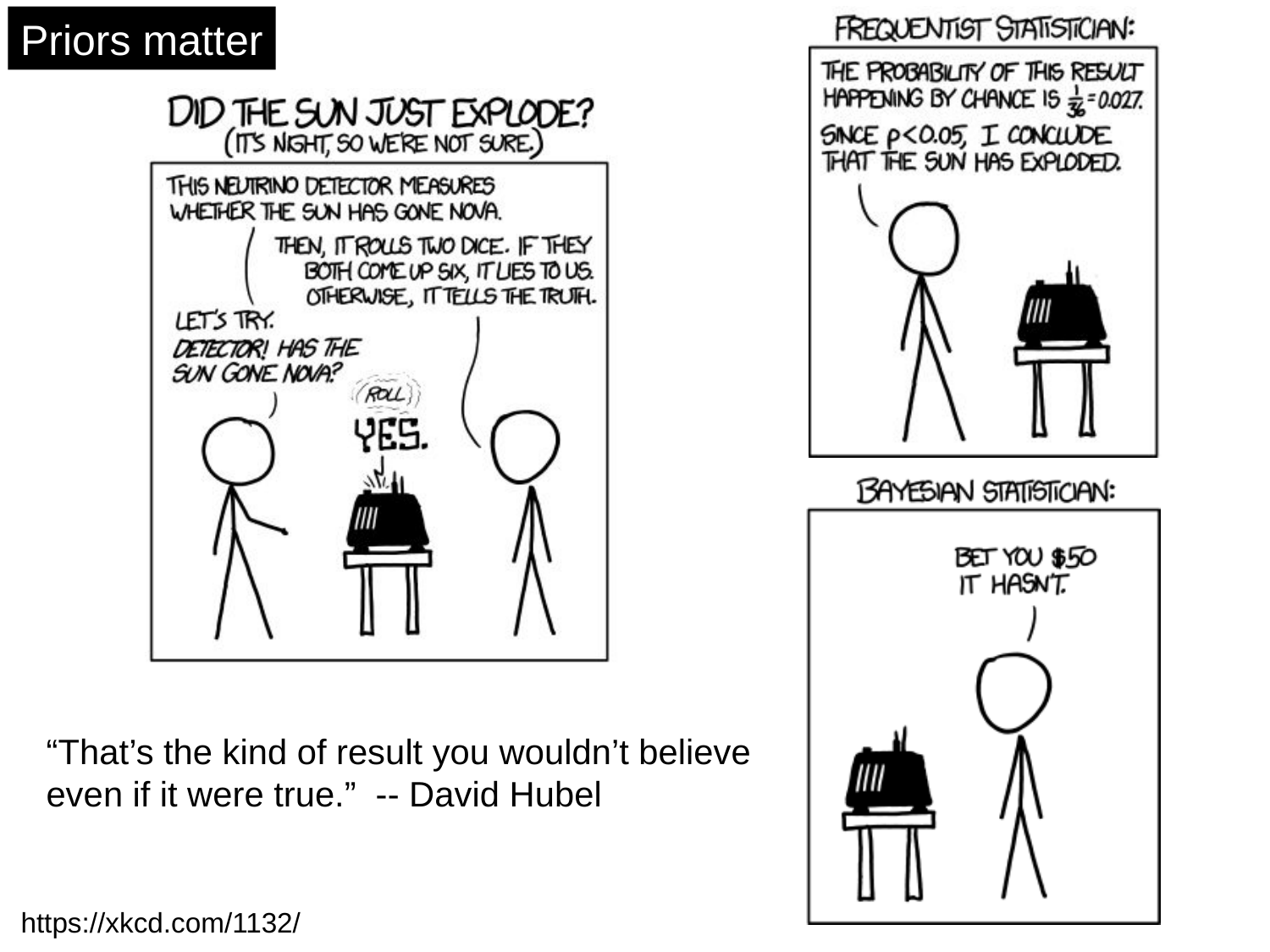

Priors matter
“That’s the kind of result you wouldn’t believe even if it were true.” -- David Hubel
https://xkcd.com/1132/

## Slide 12
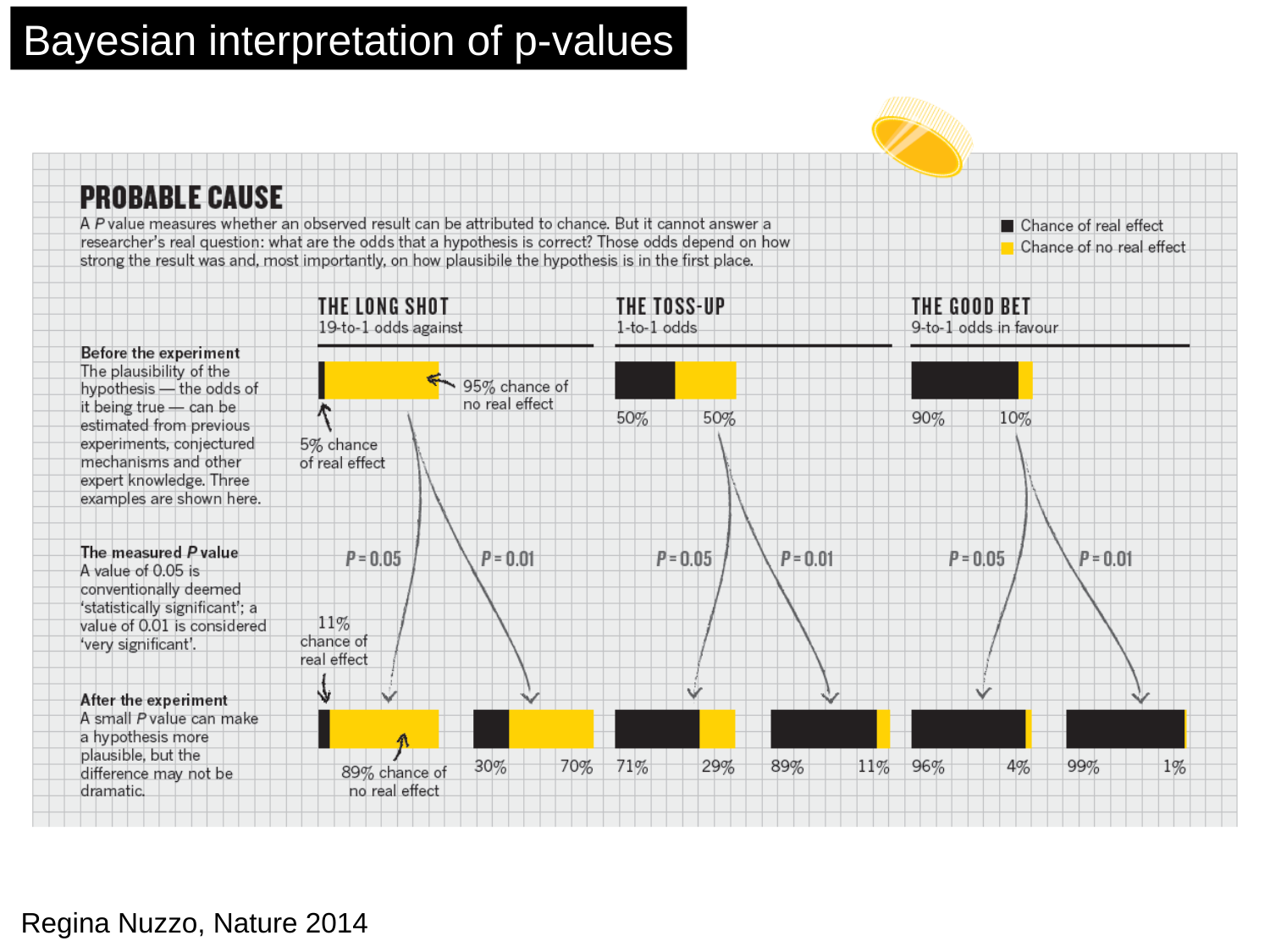

Bayesian interpretation of p-values
Regina Nuzzo, Nature 2014

## Slide 13
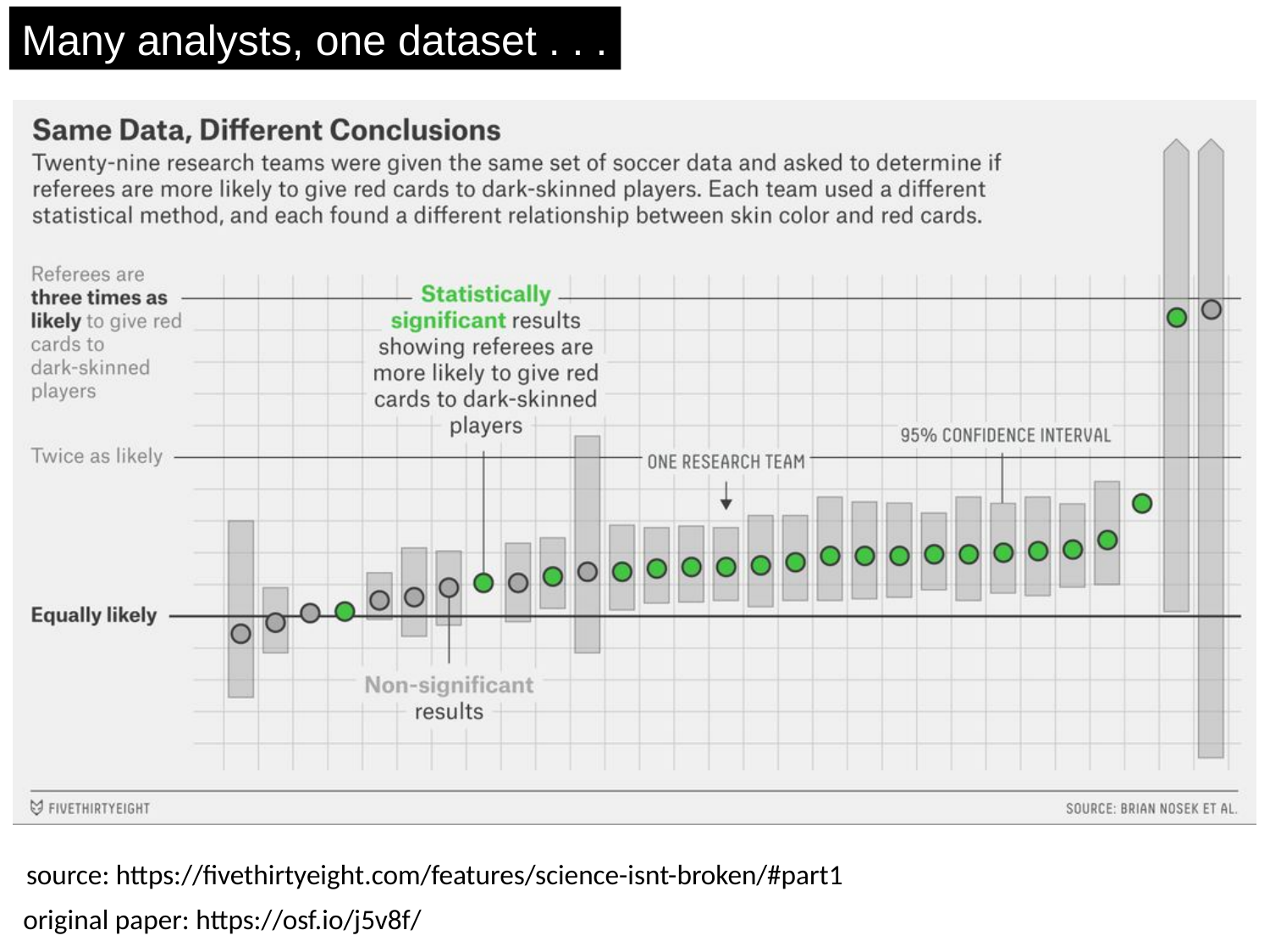

Many analysts, one dataset . . .
source: https://fivethirtyeight.com/features/science-isnt-broken/#part1
original paper: https://osf.io/j5v8f/

## Slide 14
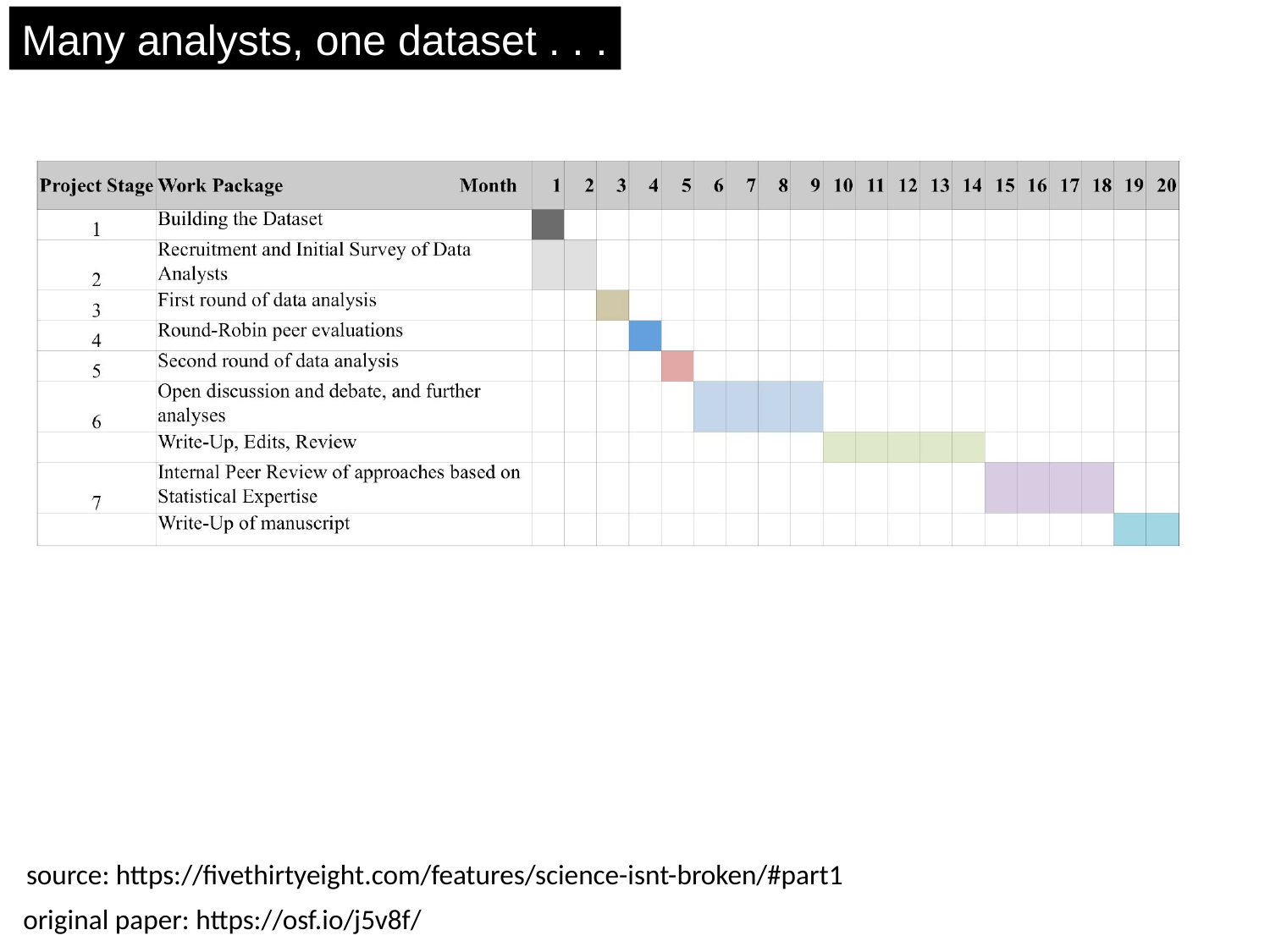

Many analysts, one dataset . . .
source: https://fivethirtyeight.com/features/science-isnt-broken/#part1
original paper: https://osf.io/j5v8f/

## Slide 15
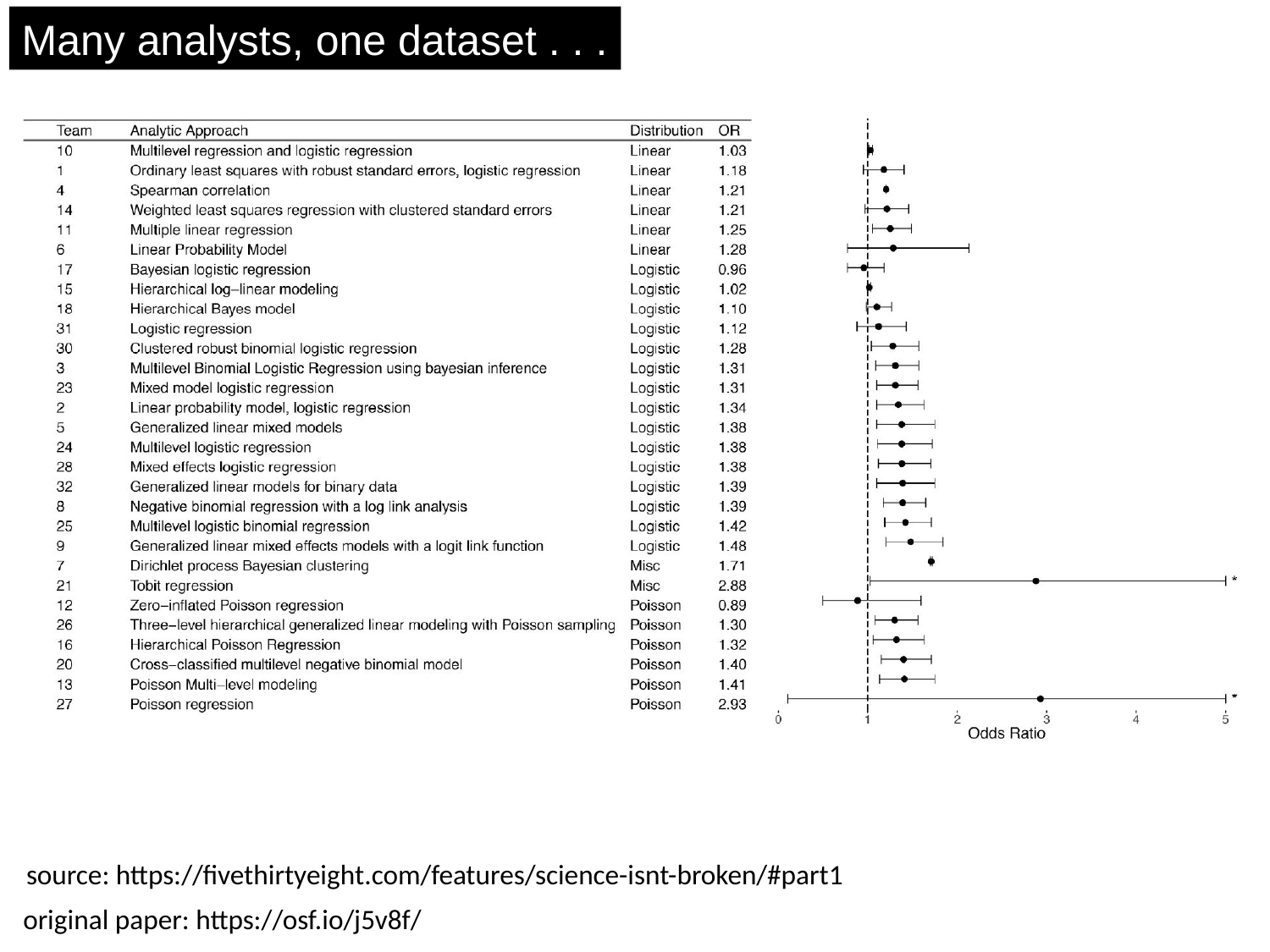

Many analysts, one dataset . . .
source: https://fivethirtyeight.com/features/science-isnt-broken/#part1
original paper: https://osf.io/j5v8f/

## Slide 16
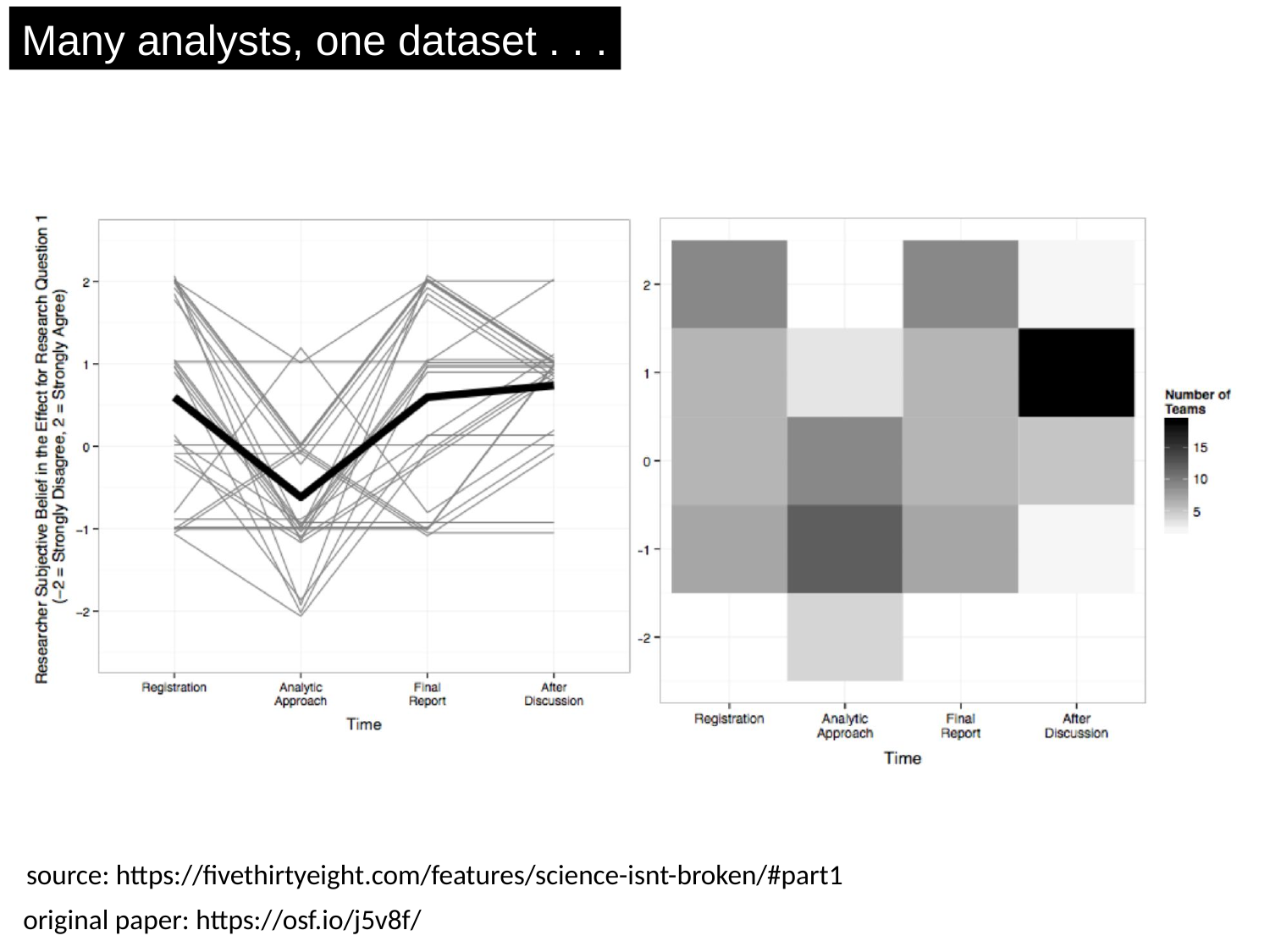

Many analysts, one dataset . . .
source: https://fivethirtyeight.com/features/science-isnt-broken/#part1
original paper: https://osf.io/j5v8f/
